# Diagnostic Tools for Autism Spectrum Disorders by Gender: Analysis of Current Status and Future Lines

**DOI:** 10.3390/children8040262

**Published:** 2021-03-29

**Authors:** Esperanza Navarro-Pardo, Fernanda López-Ramón, Yurena Alonso-Esteban, Francisco Alcantud-Marín

**Affiliations:** Department of Developmental and Educational Psychology, University of Valencia, 46010 València, Spain; Esperanza.Navarro@uv.es (E.N.-P.); M.Fernanda.Lopez@uv.es (F.L.-R.); Yurena.Alonso@uv.es (Y.A.-E.)

**Keywords:** autism spectrum disorders, diagnostic tools, gender, differential diagnostic

## Abstract

Studies on the prevalence of autism spectrum disorders have shown gender disproportion. In recent years, there has been an increasing interest in this investigation area. There are two main research lines; the first is focused mostly on gender-related biological reasons that could account for low ASD prevalence in women (i.e., related to some protective factors related to hormones or the immune system, among others), and the second research line studies possible diagnostic biases. In the present study, a review of the latter line of research is made based on two main objectives: (a) analysis of possible biases in diagnostic tools and (b) other nonbiological ASD prevalence explained by gender differences. As a result of our theoretical review, we found that the articles reviewed showed contradictory results and possible diagnostic biases, not only in their design but also in their assessment standards. We concluded that specific or complementary diagnostic tools and procedures differentiated by gender should be developed in order to reduce these biases.

## 1. Introduction

Autism spectrum disorders (ASDs) are a complex set of neurodevelopmental disorders that are defined according to the DSM-5 (Diagnostic and Statistical Manual of Mental Disorders, fifth version) [1] by two groups of symptoms: (a) communication and social-interaction deficits (CSIDs) and (b) the presence of restrictive and repetitive interests patterns, behaviors and activities (RRIBs). These symptoms appear in the early stages of development, last a lifetime and manifest in most important normal functioning areas (affective, academic, work, social, etc.), causing a clinically significant deterioration.

Evidence seems to indicate that ASDs would arise from the interaction between certain genetic lability and environmental variables, causing an early alteration in brain development [2]. One of the arguments based on the genetic basis of the disorder has emerged from epidemiological studies, showing a prevalent gender disproportion, with a higher number of cases among males in ratios of 3:1 [3], 4:1 [4,5] or 5:1 [6,7]. Differences between those studies are justified by the methodology used or by the cohort age they included, being consistent regardless of geographical origin, ethnicity, culture, etc. [8]. However, if we analyze data of prelevance according to the severity of symptoms, the differences tend to decrease [9,10]. Conversely, when the study refers to the population of high-functioning ASD (HFASD) or Asperger’s syndrome (AS), the prevalence divided by gender varies significantly, reaching ratios of 9:1 or 10:1 [11,12].

In recent years, we have witnessed a significant increase in scientific, clinical and social interest in specialized research [13] that aimed to explain the differential gender-prevalence rate in the diagnosis of ASDs [14]. In general terms, the scientific literature proposes two lines of justification. First, there is a group of research that has suggested that the low prevalence rate among females could be caused by protective biological factors. In this sense, genetic research showed results of sexual dimorphism in ASDs. Several genetic syndromes (syndromic ASDs) have been described in ASDs, which are present in approximately 10% of cases [15]. Some of the syndromes associated with ASDs seem to develop more in females (i.e., such as Rett or Turner syndrome). However, forms of ASD that are related to X-linked genes such as FMR1 (Fragile X syndrome), MECP2 (Rett syndrome) and other mutations only represent a small fraction of ASD cases, although more than 70 genes have been identified as being involved in ASDs. These data probably suggest that other X chromosome loci may play a role in the development of ASDs [15]. In this line of argument, a multifactorial theory has been postulated and proposed based on the idea that some genetic variants and environmental factors could be possibly interacting with specific characteristics of the female gender (i.e., hormones, immune function, etc.) and that they could be generating some protective factors against the development of ASDs [16,17,18,19], relating them to steroidogenesis in utero [20,21], with testosterone concentrations in the amniotic fluid [22,23] or with the excessive production of androgens (in women diagnosed with ASD) [24]. Second, there is a different research line that seems to explain the low ASD prevalence in females using the diagnosis detection bias of current assessment tools. In this sense, and precisely because of the high prevalence rates in males, Murray et al. [25] suggested the existence of ASD diagnostic-tool bias. According to Kanner [26], this disproportion of cases was presented according to gender (of 11 cases described, 8 were male and 3 were female), and they highlighted that ASDs traditionally have been seen as a predominantly male condition, and thus more attention is paid to characteristic symptoms in males [12,27,28,29,30], introducing important biases for the evaluation of females.

**Figure 1:**
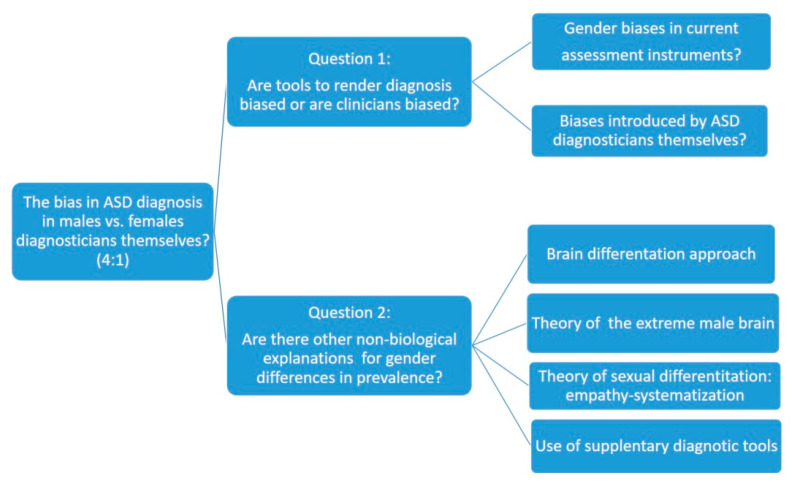
Outline of the theoretical analysis process.

The present research followed the second line of argument (i.e., it focuses the explanation of the low prevalence of ASD in females on the possible lack of sensitivity of the current evaluation instruments for detection/diagnosis). In this sense, the main objective of this theoretical review is to carry out an analysis of the possible reasons for the underdiagnosis of females according to both the nosological criteria and the characteristics of the existing diagnostic tools. To this end, we will divide the analysis into two major research questions that will guide the line of argument that we will follow for further discussion. The first research question will analyze how current diagnostic tools may bias female diagnosis of ASD, while the second will discuss complementary measures that can improve accurate ASD diagnosis in females. Figure 1 shows the proposed line of argumentation, as well as two research questions and the topics included on each case.

## 2. Analysis of Biases in Methods and Tools for the Diagnosis of ASD in Women

To answer this first research question, we will review two recent research argumentation lines that we consider of crucial importance to address the answer in a multidimensional and comprehensive way. First, we will analyze the group of studies that have focused on ASD diagnosis and the most commonly used diagnostic tools. Secondly, we will consider the group of recent studies that have assessed the differences by sex and classify their results according to the diagnostic tools used. 

Although a great research effort has been made in this investigation area, the causes of ASD are not yet known, and, at present, specific biomarkers have not been clearly identified [31]. Consequently, we believe that the ASD diagnostic process should be a result of the combination of information from multiple research areas [32]. 

In general terms, the diagnostic process can be said to begin when family members or professionals detect any of the possible warning signs. In general, the diagnostic process involves two paths; first, the confirmation of the symptoms detected as a warning signal and the level of severity of the symptoms, and second, the exploration or discarding of any known causes of that symptomatology. According to all the research studies consulted, the diagnosis of ASD can nowadays be established stably at around 20–24 months of a child’s life [33,34,35,36]. In any case, the diagnosis consists of determining whether the case we are analyzing meets the most recent and complete diagnostic criteria, as those of DSM-5 [1] and ICD-11 [37].

This diagnosis process is based on three main pillars [38]: (a) the child’s development history; (b) the symptoms´ observation and (c) the clinical confirmation. With regard to the first pillar, an interview with relatives focused on the child development history is performed, and it can be completed with the clinical and developmental data (e.g., age of appearance of the most significant developmental signs of the disorder). Second, the child’s behavior is examined, assessed by standardized tools based on the observation of child´s performance during playing games in the presence of the evaluator. Finally, after accumulating evidence about the symptoms’ presence and the consideration that these symptoms significantly affect the child´s day-to-day life and his/her family, a clinical assessment is needed to confirm the existence of an ASD.

Therefore, it is important to emphasize that ASD diagnosis represents a challenge when considering the current disorder available knowledge, not only because of the complexity of the phenotype but also because of the diversity in clinical manifestations, especially in early ages, in less severe ASD cases [39] and in more complex ASD cases that are more frequently observed in girls compared with boys [40]. Thus, many studies confirmed that ASD diagnosis for women arises later than in men [41,42,43,44], similar to other neurodevelopmental disorders (e.g., ADHD (attention deficit and hyperactivity disorder)) [45,46]. These data can be explained, in part, because in boys, symptoms are in general more disturbing with more externalizing behaviors. On the contrary, girls’ symptoms are more aligned with internalizing behaviors. These difficulties in women’s diagnoses lead to a greater vulnerability and are related to the lack of specific early ASD interventions [13] or with erroneous diagnosis. Additionally, this under-diagnosis is increased when it comes to ASDHP or AS [12,41,47].

### 2.1. Most Commonly Used Diagnostic Tools

Among the most prestigious diagnostic used tools are the ADI-R diagnostic interview (Autistic Diagnosis Interview Revised) [48,49], which has become the “gold standard” for the ASD diagnosis. The ADI-R is a clinical interview conducted with the parents or caregivers of the person suspected of suffering from ASD. The main clinical interview questions focus on three broad areas (i.e., language/communication, reciprocal social interactions and restricted behaviors and interests). The parents or caregivers’ responses are then encoded and valued using two main scoring algorithms. The first is called a “diagnostic algorithm” and assesses the complete subject’s development history. The second is called the “algorithm of current behavior”, and it scores the behavior observed in recent months. Additionally, Autism Diagnostic Observation Schedule(ADOS or its current version ADOS-2) [50,51] is a standardized protocol for observing social and communicative responses, and behaviors are coded according to specific objectives associated with each task, rating the quality and type of response [50,51,52,53]

Additionally, not only the criteria for presentation of symptoms but also the diagnosis tools validation studies have been assessed with excessively homogeneous samples (i.e., generally composed mostly with men that were Caucasian, cisgender, heterosexual and residents in the USA, Western Europe or Australia). This situation reflects a certain intrinsic tautology, given that the tools for diagnosis and understanding of ASD have been developed using samples formed mainly by men or with a low representation of women and view to demonstrate differences based on sex. 

There are other ASD diagnosis instruments that we will review as follows. CARS (Childhood Autism Rating Scale) [52,53,54,55] is a 15-item evaluation scale designed to detect and assess the ASD symptoms and other developmental disorders. CARS assesses different child development areas (social relationship, imitation, emotional response, use of the body, use of objects, adaptation to change, visual response, auditory response, use and response of taste, smell and touch, fear or nervousness, verbal communication, non-verbal communication, activity level, level and consistency of intellectual response). Each child is evaluated in these domains on a graded scale where the highest scores indicate the highest degree of deficiency. The second edition CARS-2 [55] presented two forms, the standard and another one called CARS 2-HP (High Performance). The latter was developed as an alternative measure to differentiate individuals with verbal fluency with High Performance ASD (HPASD).

The DISCO (Diagnostic Interview for Social and Communication Disorders) [56] is a standardized and semi-structured interview for the ASD diagnosis and the subsequent design of educational and treatment resources. It consists of 362 items that collect social interaction information, communication, imagination and repetitive behaviors, as well as on other aspects necessary to know the amount of support required (daily life skills, attentional difficulties, hyperactivity and challenging behaviors). The DISCO offers algorithms for the diagnosis of autism but, for its application, requires qualified and accredited personnel. 

Additionally, Baron-Cohen et al. [57] developed the Autism-Spectrum Quotient (AQ). It is a self-administered instrument composed of 50 items that measure the degree to which adults with normal intelligence have traits associated with ASD spectrum. The included features are social skills, attention shift (flexibility), attention to detail and communication and imagination, and it can be used for screening adults without intellectual disabilities (ID).

It is worth noting that the increasing concern regarding accurate ASD diagnosis has increased the number of detection and diagnostic tools, ASD identification protocols and diagnostic criteria in order to find the best description of the ASD behavioral phenotype with all possible symptom constellations. In most cases, other complementary tools such as the Social Response Scale (SRS or SRS-2, [58,59], the Repetitive-Revised Behavior Scale (RBS-R, [60]), the Vineland Adaptive Behavior Scale, 2nd Edition (Vineland-II, [61]), the CBCL (Child Behavior Checklist [62]) and the ABC (Aberrant Behavior Checklist [63]) were used. However, as far as we know, no specific or complementary tool has been developed to support women’s ASD diagnosis; thus, the possible biases in the diagnostic tools can also be reproduced in the aforementioned additional tools.

### 2.2. Differential Results by Sex According to the Diagnostic Tools Used

Frazier et al. [64] conducted a comprehensive study of 2418 participants (only 304 were women) recruited from “Simons Simplex Collection”: a core project and resource of the Simons Foundation Autism Research Initiative (SFARI), which aimed to establish a permanent repository of genetic samples from 2600 families, each with a child diagnosed with autism spectrum disorder and absence of ASD among parents and siblings. The central symptoms were evaluated by ADI-R and ADOS, and the complementary measures were SRS, RBS-R, Vineland-II, CBCL and ABC. The results indicated that women with a ASD diagnosis had a greater social communication deterioration, greater restricted interests, lower cognitive capacity, less adaptive capacity and greater externalization problems than men. The study was concluded following the line of previous work [9] in which they proposed that women that receive a correct early ASD diagnose tend to show much more salient symptoms than men, manifesting worse prognoses and worse social, communicative and cognitive functioning. 

Tillmann et al. [65] developed another multicenter study (i.e., 18 centers in nine states of the European Union) with a sample of 2139 ADI-R (376 women) and 1420 ADOS (233 women), but the study was reduced to 1030 subjects for which ADI-R and ADOS data were available simultaneously (38% of the sample). The results indicated that CR-IR evaluated in ADI-R in early childhood showed lower scores in women, while CIS levels were similar. When comparing the ADI-R and ADOS scores, no significant differences were found in the severity indexes of the disorders.

Adamou, Johnson and Alty [66] developed a study to determine possible ADOS bias in a clinical population. Out of a total of 43 participants (31 men and 12 women), they observed that the positive diagnosis rate in men was 38%, while in women it was only 25%. In addition, the obtained ASD men scores were significantly higher than the ones obtained in women, while among the undiagnosed participants the scores did not present significant differences. Although the sample was very small, the need for cut-off points or differential criteria between men and women was emphasized.

Due to this, we believe that, as pointed out by Lai et al. [13], current diagnosis methods that are perhaps excessively dependent on ADOS and ADI-R may show low detection sensitivity of the behavioral ASD female phenotype; therefore, complementary measures have been pursued to improve sensitivity in the diagnosis of female ASD.

Kumazaki et al. [67] applied CARS to examine gender differences by comparing 20 girls and 20 boys aged 5–9 years and diagnosed with ASD/HF. They observed that boys scored significantly higher on “body use”, “use of objects” or “activity level”, while girls scored higher on aspects of sensory sensitivity (taste, smell and touch). If this differentiation is confirmed, this instrument could be an eligible candidate for complementing girls’ early diagnosis.

Duvekot et al. [68] applied SRS-2-child as an ASD screening tool in general mental health centers for children and adolescents. They observed that the male–female ratio among the subjects diagnosed was 2.6:1, while with a later confirmed diagnosis the ratio varied significantly, at 3.7:1. These ratios suggested that women that show symptoms in the SRS screening tool are less likely to meet the diagnostic criteria for ASD even following rigorous standards of good practice. In this sense, it is possible that there could be a nosological problem underlying the current ASD concept that would not take into acocunt the group of female autism manifestations. 

Ratto et al. [69] also developed a multicenter study out of a total of 816 participants (125 women) who did not have an ID (intellectual disability) and met the diagnostic criteria for ASD by ADI-R, ADOS or both. Participants were selected by age and IQ and by using a random procedure to obtain two equal samples constituting two groups (114 men and 114 women) in which the scores in both diagnostic instruments did not show significant differences according to sex. In their study, the authors introduced two complementary measures (i.e., SRS Vineland-II). The differences found according to the scales used should be achieved in relation with the scale’s nature. In this sense, it is worth noting that the SRS is completed by parents or primary caregivers using a Likert scale and, unlike ADOS and ADI-R, is standardized on the basis of sex, as the authors identified critical sex differences during the validation of this questionnaire. The scores are generated from the assessment of the five domains of the ASD traits (social awareness, social cognition, social motivation, social communication and restricted/repetitive behaviors), indicating higher scores and higher levels of autistic traits divided by sex. On the other hand, the Vineland-II is a scale that evaluates adaptive skills in individuals from 0 to 90 years old, dividing adaptive behaviors into three domains: communication skills, daily living skills and social skills. Since both SRS and Vineland-II are measures standardized by sex, it worth noting that ASD scores in women were assessed separately in this diagnosis tool (i.e., by comparing them to typically developing women and not with male samples), and they were shown to have more severe symptoms than autistic men when compared to typically developing men. As a result of this study and the confirmations that were found in previous research [66,70], it can be concluded that any diagnosis that is excessively dependent on the scores of ADOS-2 or ADI-R could be clearly carrying gender bias consequences.

In a recent multicenter study [71], a total of 8982 cases (1463 women) were collected, consisting possibly of one of the largest samples of women evaluated for ASD symptoms to date. Information from the ADI-R, ADOS and SRS was available, and a parametric integrative random mixed-effect analysis model was constructed in which the sample was balanced by age, IC and language level. The results do not demonstrate the existence of significant differences in severity levels evaluated by these tools; thus, the authors recommended as future lines of research to focus on possible false negatives, especially in women, who are generally diagnosed later. This approach requires complex, high-cost follow-up case studies. In addition, there is growing evidence that the diversity of both gender identity and sexual orientation is not only present in autism but, in fact, occurs in higher rates among autistic people [72].

We believe that one of the problems we face is precisely the lack of a explicitly defined behavioral phenotype for ASD in general (level of cognitive functioning, linguistic ability, learning ability, etc.) and, in particular, of the female phenotype and its evolution throughout personal development [73]. It is worth noting that there is an additional group of characteristics or additional behaviors (i.e., in relation to the symptoms described in DSM 5 or ICD 11) that we think that should be taken into account when assessing ASD disorder in women, and, even when they cannot confirm the diagnosis, it could be desirable to determine a protocol to be followed in order to rule out false negatives in women’s ASD diagnoses. In this sense, the reviewed results on the use of standard tools such as ADOS and ADI-R are still unclear, and they require complementary information provided by tools (i.e., such as SRS and or Vineland-II) that can possibly improve the sensitivity of women’s ASD detection.

## 3. Other Theoretical Approaches

In order to answer the main research question of the present work, we have come across a body of information that, in many ways, is not conclusive and even reveals contradictory aspects. Therefore, and to deepen the theoretical review, in this section we will focus on reviewing the existent evidence and the conceptual theories that would allow us to envision a more systematic and coherent theoretical alternative.

Is it worth mentioning the study conducted by Lockwood-Estrin and collaborators [74] in which they carried out a systematic study to determine the possible intrinsic difficulties that could be the basis for the existing biases in women’s ASD diagnosis (i.e., in girls and young women under 21 years of age). They identified two main groups of possible difficulties. First, they found that the group of symptoms and behaviors used for diagnosis (e.g., RRIB and CSID) are highly masculinized. Second, they observed that there are other diagnosis barriers such as a bias in evaluators or observers when considering, by training or deformation, that ASD is mainly a male disorder.

Regarding the first groups of difficulties, and regarding RRIB symptoms, it is worth mentioning that in some cases a greater weight is attributed to repetitive behavior patterns and restricted interests in males [9,75,76,77,78,79,80]. These contradictions may be due to the so-called camouflage skills, most frequently shown in women [28,47]; this skill can be developed by learning, and there is no evidence supporting its presence in young girls compared to in youth and mature women [43,81]. A second justification in the underestimation of RRIB in women can be found in the biases of the evaluator; certain behaviors or restrictive interests of women, such as the interest for celebrities or self-image, cosmetics, etc., are perceived as more normal than other restricted male behaviors or interests [12,44,76,82], while before the age of six there are no differences [30].

Regarding the CSID group of symptoms, some studies indicate that girls score higher in social skills than boys [75,83], while others show an opposite pattern [64]. Another group of studies completely denies any gender difference [12,13,30,76,77,83]. Additionally, recent studies showed the existence of a gender-differentiated profile in aspects related to oral expression [81,82,83,84,85,86].

Due to the abovementioned factors and as a consequence of the contradictions found in the possible differences in gender in ASD, some other promising research lines have been proposed beyond the symptoms usually used for women’s ASD diagnosis. In this regard, four main theoretical approaches can be distinguished in the literature: (a) the approach of brain differentiation, (b) the theory of the extreme male brain, (c) the theory of empathy-systematization sexual differentiation, and (d) the approach based on the use of complementary evaluation questionnaires. Next, we will explain each one of them, considering them a possible theoretical contribution that can constitute the basis for a possible effective tool of differential diagnosis by gender in ASD. 

### 3.1. The Approach of Brain Differentiation

Regarding the theoretical approach based on brain differentiation, it was proposed that cognitive differences between men and women could explain why men were more likely to suffer from ASD. Although there are evident differences in the manifestation of ASD according to sex, which would probably indicate the existence of a female neuropsychological profile of autism, there is no agreement on what characteristics would make up this ASD profile [30]. Due to this, it is necessary to develop differential tools to detect the specific symptoms in men and women or, at least, differential scales using sufficiently large and representative samples of both sexes.

### 3.2. The Theory of the Extreme Male Brain

The theory of the extreme male brain [87] postulates that autistic people would score, on average, towards more “male” positions [88]. The so-called “Reading the Mind in the Eyes” test is an instrument [89] derived from this approach which has been widely used to measure theory of mind (ToM) [90] or the ability to recognize the thoughts and feelings of others; this test was applied in adults with autism (395 adults with autism, 178 men and 217 women; and 320 control adults, 152 men and 168 women), finding that, although some parts of the test reflected social difficulties that happened to be common to both sexes, the differences found between the groups fitted the extreme male brain hypothesis. In addition, the performance of women with autism differed more from that of same-sex controls than that of men with autism, and their self-assessment of symptoms was more consistent with test results than other groups.

### 3.3. The Theory of Sexual Differentiation Empathy-Systematization

The theory of sexual differentiation grounded on empathy-systematization (E-S) abilities [91] postulates that, in the general population, people can be classified based on their score in these two dimensions. In this sense, empathy is understood as the impulse to recognize another person’s state of mind and respond with an appropriate emotion. Systematization is defined as the impulse that a person can be shown to analyze and build a system that follows rules or patterns. A recent study [91] that was carried out on a sample of more than 670,000 participants, including more than 36,000 subjects with autism, attempted to demonstrate the theory of E-S sexual differentiation, finding that male patterns tended to score high on the D scale (difference between systematization and empathy); a high D score was related to men, while a low D score was typically obtained in women. It is necessary to point out that empathy has two main forms or components: cognitive (the ability to recognize another person’s thought or emotional state) and affective (the emotional response appropriate to the other person’s feelings). Autistic subjects would score lower in cognitive empathy by showing a deficit in mind theory but not necessarily in affective empathy. Additionally, women with ASD would score higher than average men on interpreting themselves to be generally hypermasculine, although they do not manifest other masculine traits such as aggressiveness. 

### 3.4. Use of Complementary Evaluation Questionnaires

Finally, there is a theoretical line that proposes the use of complementary evaluation questionnaires for evaluating the main symptoms of ASD-related constructs. For example, the Friendship Questionnaire (FQ) was used in a sample of 68 adults (51 men and 17 women) diagnosed with ASD [92] showing that the two sub-samples (i.e., male and female) scored significantly lower than healthy controls, but also that friendship relationship styles differed by sex, thus additionally supporting the theory of the extreme male brain.

To sum up, we believe that these four general explanatory approaches could be useful in finding a possible diagnostic plot line that would allow generating appropriate theoretical bases to create an effective and more accurate ASD diagnosis tool that can be specific both for women and for men.

## 4. Discussion and Conclusions

The theoretical review carried out in this article showed that in recent years there has been an increase interest in the female phenotype of ASD characterization. We believe that the reason for this interest lies, on the one hand, in determining whether there is any protective factor that could be identified, and, on the other hand, whether the problem is grounded in the diagnostic instruments or in the use of them. As it was argued in the present work, to date the studies consulted clearly provide inconclusive results.

We think that is worth mentioning that it is important to distinguish between sex and gender in the context of autism. Sex is a biological or physiological characteristic, while gender is a socio-cultural construct [93]. Due to this, gender could be defined as the combination of biological sexual characteristics with factors related to behavior, social roles, lifestyle, life experiences, etc. [94].

In addition to the possible bias introduced by clinicians and researchers, the most widely used instruments for the diagnosis of ASD (ADI-R and ADOS) also present a certain bias when assessing ASD traits in women. They were built on the nosological concept that ASD were male disorders and, therefore, tend to evaluate more male conditions. In addition, the scales that are usually used to determine the cut-off points of each instrument have been also performed on gender-biased samples. All this leads us to believe that, regardless of the possible female phenotype of ASD, it is necessary to investigate more towards the development of analytical tools or complementary instruments for a proper ASD diagnosis in women. The evaluation instruments reviewed in the present article allow us to suggest that ASD in women, and especially those with high functioning ASD (HFASD), are in a very vulnerable situation as they go unnoticed and cannot access the care services and therapies that they need. In addition, they normally are not exempt from stereotypes, gender roles and social expectations (being affectionate, pleasant, friendly, sociable, etc.) which adds an additional stress burden apart from all of the abovementioned. As a result, these women may feel misunderstood, with a greater social pressure to be normal and with more internalizing problems (i.e., especially anxiety and depression) and with fewer opportunities to access health, educational and social services that would help them improve their quality of life [94].

Additionally, the common comorbidity of mental disorders in women with HFASD poses a double problem. On the one hand, by failing to detect and diagnose HFASD, they are considered to show a neuro-typical development and are normally removed from early intervention programs. On the other hand, they are normally diagnosed with another disorder, or they develop an additional condition as a consequence of suffering sustained stress and anxiety.

Based on the theoretical review carried out in the present article, two possible lines of research are proposed for future research. First of all, regarding ASD diagnostic tools for women, we believe that they should be improved by including not only the DSM-5 criteria, but also other variables which recent studies have shown to be present in HFASD (i.e., such as socialization styles, friendship relationships, looking patterns and looking avoidance, type of speech, etc.). Secondly, regarding the reviewed theoretical approaches (i.e., that could sustain an explanation for the lower prevalence of HFASD diagnosis in women) we found that together they can be considered for a possible effective gender differential diagnostic tool in HFASD, and they could be useful to create an effective HFASD gender differential diagnostic tool. Due to this, we consider that another research line could arise (i.e., by means of properly validated and assessed questionnaires) from evaluating aspects of female HFASD (i.e., different from those described by the possible extreme male brain) and based mainly on women-specific traits (e.g., such as those studied in the empathy-systematization approach) and assessing possible intervening co-variables (i.e., such as those described in the section on the use of complementary evaluation questionnaires). A final line of future work will be the incorporation of digital means in the diagnostic process, so that it will be feasible through machine learning elements to improve the prediction and diagnostic adjustment in both men and, specifically, in women.
